# Unification of Epistemic and Ontic Concepts of Information, Probability, and Entropy, Using Cognizers-System Model

**DOI:** 10.3390/e21020216

**Published:** 2019-02-24

**Authors:** Toshiyuki Nakajima

**Affiliations:** Department of Biology, Ehime University, Ehime Prefecture 790-8577, Japan; cognizers@yahoo.co.jp; Tel.: +81-89-927-9639

**Keywords:** cognition, cognizers system, information, probability, entropy, observer, observation, (un)certainty, relative frequency

## Abstract

Information and probability are common words used in scientific investigations. However, information and probability both involve epistemic (subjective) and ontic (objective) interpretations under the same terms, which causes controversy within the concept of entropy in physics and biology. There is another issue regarding the circularity between information (or data) and reality: The observation of reality produces phenomena (or events), whereas the reality is confirmed (or constituted) by phenomena. The ordinary concept of information presupposes reality as a source of information, whereas another type of information (known as *it-from-bit*) constitutes the reality from data (bits). In this paper, a monistic model, called the cognizers-system model (CS model), is employed to resolve these issues. In the CS model, observations (epistemic) and physical changes (ontic) are both unified as “cognition”, meaning a related state change. Information and probability, epistemic and ontic, are formalized and analyzed systematically using a common theoretical framework of the CS model or a related model. Based on the results, a perspective for resolving controversial issues of entropy originating from information and probability is presented.

## 1. Introduction

Science has been increasingly diversified into various disciplines or research fields, creating many concepts and terms used with special meanings inherent to each field. However, very few fundamental concepts are shared by almost all of them. “Information” and “probability” are such concepts, and these terms play wide and essential roles in scientific investigations. Science uses two types of fundamental language: Epistemic (from Greek epistēmē, meaning “knowledge”) and ontic (from Greek ōn, ont-, meaning “being”). Epistemic language concerns how to know (understand) about object things or processes; ontic language concerns the object things or processes. Several essential terms are shared in the sciences, such as: “Observer/observation”, “measurement”, “data”, “phenomenon”, “event”, and “[un]certainty” for the epistemic field; and “system”, “measurement device”, “matter/energy”, “state”, “change”, “interaction”, “process”, and “pattern” (“form”, “structure”, “configuration”, “[dis]organization”, and “[dis]order” as related terms) for the ontic field. 

The concepts of information and probability involve both epistemic and ontic fields under the same terms; epistemic and ontic are sometimes referred to as “subjective” and “objective”, respectively, for these concepts. Probability has two meanings: The degree of the certainty of an event occurring, as the epistemic concept, and the relative frequency of an event (or state) occurring, as the ontic concept [1]. Hacking [2] called this duality as “Janus-faced”. Information is also used to mean knowing (or knowledge, data) as an epistemic concept, and pattern-transmission as the ontic one [3,4]. Dictionary definitions of the term “information” are given as (1) facts provided or learned about something, (2) what is conveyed or represented by a particular arrangement or sequence of things [5]. The term “inform” etymologically means: *Form the mind to*, *describe, to give form or shape to* (*ibid.*), including both epistemic and ontic processes. For example, sequence data of a DNA molecule is epistemic information for biologists who want to understand a particular process of life. Such DNA molecule can also act as ontic information within a living cell when its sequence pattern is transmitted to another pattern of an amino-acid sequence of a protein. Concepts of entropy and of the amount of information also inherit these dual meanings of probability and information [6].

This duality of epistemic and ontic (or subjective and objective) fields (the E-O duality) in these concepts produces conceptual and theoretical problems when they are used under the same mathematical formalisms. For example, controversy exists as to whether entropy in physics represents a quantity of a subjective state of knowledge or a quantity of objective properties about an observed system [7,8]. In addition, there is another aspect of the issue of E-O duality: When an observer is a material entity as a member of a material system and not an epistemic entity outside the system. This aspect is easily understood by considering that scientists are also material entities as humans who can interact with object systems, social or natural, under investigation (e.g., observation of animal behavior, experiments in quantum physics). In biology, living entities, such as animals, plants, and microbes, are all material entities functioning as subjects of knowing through their neural and/or inter- or intracellular signal molecule processing of information within an observed system such as an ecosystem. Here, “knowing” by organisms is not an epistemic but rather a material process that is evident in behavioral and brain sciences. Immune networks can also work as cognitive systems, like brains, in which the production of an antibody promotes or suppresses the production of other antibodies, generating a network of cellular-level events [9,10]. The E-O duality suggests knowing (observation) and material changes (such as movements, action) have not yet unified into a single concept, according to which, information and probability theories have not yet unified in a monistic framework. In this paper, I describe such a monistic model for information and probability, called the cognizers-system model (the CS model), in which observations (epistemic) and physical changes (ontic) are both described as cognition, defined as “state change in relation to other entities”; put simply, “related state change”. 

Another fundamental issue exists in theorizing information and probability. The above argument is based upon the initial assumption that the external reality (or universe) exists as it is. However, it is unclear whether data are caused by an observed system that exists independent of observation (i.e., the ordinary type of information), or data create something real (i.e., *it-from-bit* type of information), which is a difficult issue in philosophy [11,12,13], physics [4,14,15,16,17,18], and biology [19,20,21,22]. This issue may be called “the circularity of entailment between epistemic and ontic fields” (denoted “the E-O circularity”). This circularity, like “the chicken or the egg” problem, occurs as follows: Observation of the reality existing independently of a subject produces phenomena (or events) in the subject, whereas the reality is realized (constituted) from phenomena. In other words, observation presupposes the existence of reality outside the observer, whereas the reality is confirmed by observation. This circularity has been recognized historically, which is clearly represented in a fragment by Democritus: “The intellect says: “Ostensibly there is color, ostensibly sweetness, ostensibly bitterness, actually only atoms and the void”; to which the senses retort: ”Poor intellect, do you hope to defeat us while from us you borrow your evidence? Your victory is your defeat” [14] (Chapter 6). In quantum physics, information can be understood as data or distinction from which physical reality is made; it-from-bit by Wheeler [16] is this kind of information. Nakajima [23] proposed an internalist model for explaining how the external reality can be “realized” (or “to become real”) from the phenomena by the subject (the self, the mind). This kind of information, as realization, is important in science in which scientists try to constitute natural/social reality from data (phenomena) and in understanding living systems. Scientists and living systems, in general, cannot go outside themselves. All they are capable of is processing data, creating (models of) reality, and acting; in principle, they cannot determine whether the data originate from real things that exist outside.

To avoid confusion from terminology, let us use the term “observation” of something as to know something that is presupposed to exist, whereas “realization” of something is knowing something as a construct by the subject; “knowing” is used as a collective term to mean either of them. Therefore, the E-O circularity teaches us that knowing (or knowledge) can have two different meanings in this context: To observe (observation) and to realize (realization: Making it real). As represented in the E-O duality, the epistemic concept of information indicates knowing (or knowledge, data). According to the two meanings of knowing (i.e., observation and realization), there are two epistemic information concepts: The information as observation and the information as realization. The former is usually used in information science founded by Shannon and Weaver [24], whereas the latter is usually called it-from bit information in contemporary physics [4,16].

Presumably, no one doubts the importance and roles of the concepts of information and probability and derived or related concepts, entropy and the amount of information, in promoting scientific investigations. However, the E-O duality together with the E-O circularity produces a complicated situation of information-theoretical approaches in science, particularly biology. In this paper, I attempt to provide clear definitions in a single, monistic framework, called the cognizers system model, and explain the relationships between them. In this endeavor, two types of world modeling are distinguished: One is the internalist model in which only a subject entity is given and its environment (or the external world) is derived within the subject (“internalist model” is different from “internal model”; see Section 7.1). The other is the externalist model in which the world and its material elements (entities) are given (defined) as a hypothesis and described from outside. The former model is more conservative than the latter because its modeling is based on data or percepts that the self has and no entity is assumed.

## 2. Overview 

The major aim of this paper is to present a monistic framework of information and probability theories. First, from an externalist point of view, I describe the cognizers-system model (hereafter, the CS model) for unifying epistemic and ontic languages by use of the concept “cognition” as a state change in relation to other entities. The term “cognition” in the CS model is different from common usage of the term, where it is used exclusively for the epistemic field in the ordinary usage, whereas it is used for both epistemic and ontic fields in the CS model. In this paper, I first overview the CS model and the extended concept of cognition (Section 3): The cognition concept unifies the epistemic state-changes in observation for a mental subject and the ontic state-changes for a material subject as state-changes of an entity of the same kind. This extended usage can resolve the issue of E-O duality in the framework of the CS model. Both epistemic and ontic entities are called “cognizer”, which is a subject of state-changes in relation to other cognizers, i.e., interactions, in a cognizers-system. Then, I address the E-O duality in information and probability concepts. “Information” is defined as the related state-change. Therefore, “information” has the same meaning as “cognition” in the model. Thus, epistemic information and ontic information are conceptually unified and explained in the same language, i.e., the CS model. The relationship between various types of probability concepts, including the degree of certainty and relative frequency, as well as entropy and the amount of information, are formalized and explained within the same framework of the CS model. In particular, three types of observer, the meta, external, and internal observers are distinguished for theorizing. The meta-observer is the model builder of a CS model as the world. External observers exist outside observed sub-systems. Internal observers are system components of an observed system.

Secondly, from an internalist point of view, I address the issue of E-O circularity using a more conservative model in which only a temporal sequence of data or percepts are assumed, and I seek for an algorithm that can derive foreign elements that do not belong to the given sequence. The derived elements are formed within the subject, e.g., in the downstream of the sequence or another. The algorithm I propose is called inverse causality, the contraposition of the statement of the principle of causality. Inverse causality corresponds measurement (distinction) of different states of the reality in the above externalist CS model. This argument concludes that quantum physical measurements, represented as it-from-bit information, assume the inverse causality that is equivalent to the deterministic world model (i.e., the principle of causality). If the subject can perform this kind of algorithmic processing, it constitutes an internal model for the external reality. From the view point of the meta-observer (builder of the internalist model), many such subjects can exist—a subject cannot look over a population of subjects including itself due to the incapability of going outside of itself. From this viewpoint, their internal models can vary depending on the entire dataset that each subject has, and on partial data chosen from the entire dataset and used for derivation. This variation of internal models about external reality can explain the diversity of umwelt in living systems in an ecosystem. In science, a variety of world views can exist depending on the subject.

Lastly, I attempt to unite the externalist and internalist models into a single theory. The major idea is that the externalist model is built within a subject. In other words, the externalist model is a kind of internalist model that is built within a subject for realizing reality from phenomena occurring to the subject.

During this course of the argument, I review publications concerning the above models and then describe an entire synthetic framework toward a unified theory. Using the internalist and externalist models, several types of information and probability concepts are defined and explain how they are related to others.

## 3. Externalist Model of the World and Systems: Cognizers-System Model

### 3.1. The World, Systems, and Cognizers 

#### 3.1.1. Overview

**Figure 1:**
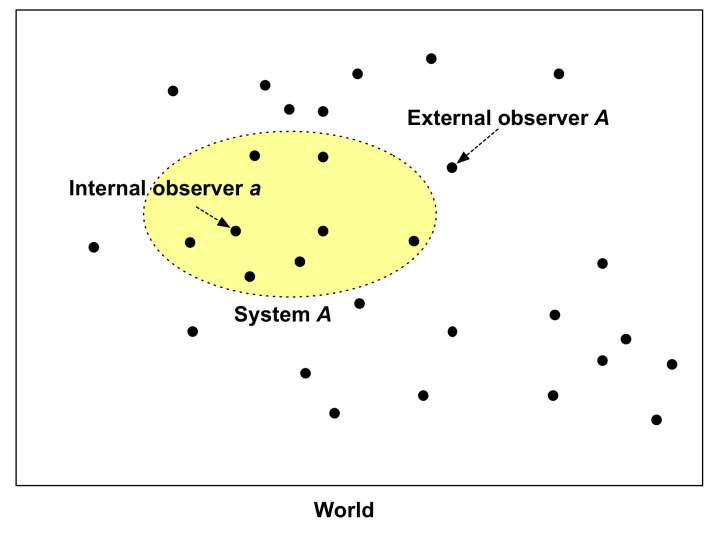
Externalist model of the world using the cognizers-system model (CS model). The meta-observer describes a model of the world (squared area). Dots denote cognizers in the world. The world is the whole cognizers system that can harbor partial systems (e.g., system *A*). There are two types of cognizers functioning as observers: External and internal observers. External observers, e.g., external observer *A*, do not belong to the system they observe, whereas internal observers, e.g., internal observer *a*, belong to the system they observe.

The meta-observer describes a model of the world as the whole system of cognizers, i.e., the CS model. The whole cognizers system (the world) is a metaphysical construct, which is a model of the world. Part of the world can be observed as a system, which is a collection of interacting cognizers (inter-cognition). A variety of partial cognizers systems (e.g., system *A* in Figure 1) can be harbored in a nested-hierarchical way or another in the world. There are two types of cognizer functioning as observers: External and internal observers. In the CS model, cognition and cognizer are general terms that include observation and observer as special kinds of cognition and cognizer, respectively, which are used dependent on the context. The term observer is usually used for a cognizer that has a memory as an internal structure, and observation is used for memory-involving cognition. External observers do not belong to the system they each observe (e.g., external observer *A* in Figure 1), whereas internal observers belong to the system they each observe. Whether a given cognizer (observer) is “external” or “internal” (identically, outside or inside, respectively) to a particular system does not depend on their location in the physical distance space; instead, it depends on the membership to the system as a component. The meta-observer exists nowhere and nowhen within the world; it rather exists at the meta-level of the world-as-a-model and knows the world as an omniscient entity. 

#### 3.1.2. External Cognizers (Observers)

Any set of cognizers within the world (i.e., the whole cognizers system) can potentially form a partial system (e.g., system *A* in Figure 1). Hereafter, “system” is used instead of “partial system” when not confusing. Cognizers outside a focal system comprise “the environment of the system”, which is the rest of the world. An external observer, which may include a measurement device, belongs to the environment of the observed system. The system boundary is arbitrary in modeling. However, in science, the boundary is chosen such that the system behaves approximately deterministic. This is possible when the system is delineated such that the environment of the system is nearly constant to the system. Notwithstanding such delineation, any system within the world is not deterministic in a strict sense because events occurring outside, including acts of observation, may cause uncertain behavior of the system through interactions, even though very weak, with entities outside the system. However, as is often seen in science, it is possible that theoretical models for those partial systems behave deterministically by assuming their environments are constant or controlled by a certain deterministic rule.

#### 3.1.3. Internal Cognizers (Observers)

An internal observer belongs to an observed system as a component. Internal observers do not observe entirely the partial system to which they belong to (e.g., system *A* in Figure 1); instead, they each observe their environments (the rest of the system) that interact with them—this environment should not be confused with “the environment of the system”. For example, when you observe a person in conversation with yourself, your observation occurs internally in the two-person system, consisting of the person and you. The person you observe is your environment, and this environment also observes you. 

### 3.2. Cognition in Cognizers-System Model

Definitions of cognition and cognizer are as follows: Cognition is a determination of a particular state in relation to states of others. The determination involves two fundamental properties of cognition: (1) Discrimination between different states of others, with discriminability as its ability; (2) selecting one particular state among many possibilities, with selectivity as its ability. The entity that performs cognition is called a cognizer. A cognizer is a material, or subject entity, that has a particular state at each moment and changes its current state to another state, including non-change (i.e., a change to the same state, as a special case), depending on the states of cognizers in the environment. The cognition concept in the CS model includes any state-change of an entity (cognizer), including the acquisition of data by an observer and movements of physical entities. Formally, a cognizer *C* is defined with its own state-space, **C**, and the property that determines its state-change, *f_C_*, in relation to others, as defined above. 

**Figure 2:**
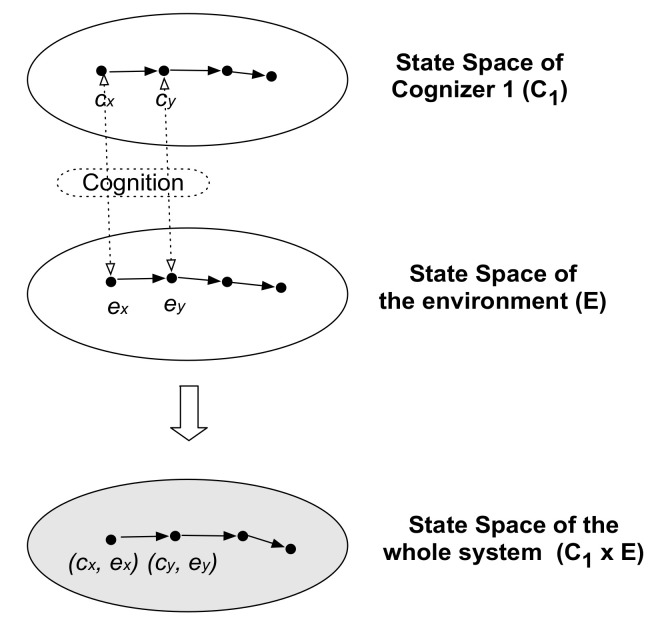
A two-cognizer system composed of a focal cognizer *C_1_* with state space **C_1_** and its environmental cognizer *E* with state space **E**. The environmental cognizer may be composed of many cognizers such as *C_2_, C_3_,* …, *C_n_*. Arrows indicate temporal state-changes of component cognizers by cognition.

The world is composed of cognizers. The world, the whole cognizers system, is a deterministic system in a discrete time unit. A two-cognizers system, for example, consists of two cognizers forming the world (Figure 1). Being composed of only two components does not necessarily mean the system is simple. It is possible, for example, to take a particular atom as one cognizer, and take the rest of the universe as another cognizer; this is not simple. Figure 2 shows a two-cognizers system composed of a focal cognizer *C_1_* with state space **C_1_**, and its environmental cognizer *E* with state space **E**. The environmental cognizer may be composed of many cognizers, such as *C_2_, C_3_,* …, *C_n_*. Arrows indicate temporal state-changes of component cognizers by cognition, which is formalized as *f_C1_ (c_i_, e_i_) = c_j_; f_E_(c_i_, e_i_) = e_j_* (*i = x, j = y* in Figure 2), where *f_C1_*: **C_1_** × **E** → **C_1_** and *f_E_*: **C_1_** × **E** → **E**, where italicized capitals are used to denote cognizers, and bold-faced capitals denote their state sets; the arrow (→) indicates mapping by the function *F,* as follows. The state transition of the whole system is given as

…, *(c_i_, e_i_), (f_C1_, f_E_)(c_i_, e_i_), (f_C1_, f_E_)^2^(c_i_, e_i_)*, …,

where *(f_C1_, f_E_)(c_i_, e_i_)* is defined as (*f_C1_ (c_i_, e_i_), f_E_ (c_i_, e_i_)),* which is simply denoted as *F(c_i_, e_i_),* where *F* is the motion function or the whole system. Therefore, we obtain

…, *(c_i_, e_i_), F(c_i_, e_i_), F^2^(c_i_, e_i_),* …,

where *(c_i_, e_i_)* is the state of the whole system *U* with the state space **U** (*F:* **U** → **U**). Therefore, we obtain

…, *u_i_, F(u_i_), F^2^(u_i_),* …,

where *u_i_* = *(c_i_, e_i_).* The CS model is deterministic by *F* (Appendix A).

From the meta-observer’s viewpoint, “cognition” is defined as a state change of a focal cognizer in relation to the current state of the environment. Suppose that a focal cognizer changes its state from *c_i_* to *c_j_* when the environment state is *e_i_*. From a cognizer viewpoint, this cognition (c_i_ → c_j_) occurs in relation to the environment (state *e_i_*), which can be interpreted as an “event” for the cognizer, which experiences the environment by observation (cognition); the arrow (→) indicates a state-change. Each state is not an event for cognizers; “state” is a meta-observer’s language. This aspect of cognition corresponds to an epistemic representation of information, as described in detail below. From the viewpoint of the meta-observer or an external observer, cognition *c_i_* → *c_j_* occurs in relation to states of other cognizers in the system. In this sense, cognition is a related state-change. This aspect of cognition corresponds to an ontic representation of information (i.e., related state-change), as described in detail below. The related state-change includes unrelated state-change as a special case; i.e., non-discrimination between different states of environmental cognizers. 

## 4. Cognition and Information 

In this section, I attempt to resolve the issue of the E-O duality in information and probability concepts, by using the concept of “cognition” in the CS model, in which epistemic knowing and material movements, including movements of physical particles, are both represented as a cognition by cognizers. The difference between epistemic and ontic fields depends on the types of cognition and/or the cognizer under consideration.

### 4.1. State and Event

In the CS model, state and event are different concepts. States are defined by the meta-observer (MO), who can discriminate every state from others in the CS model. Any cognizer inside the world cannot identify the states of the object cognizers directly; instead, it knows them by cognition; cognition may be interpreted as observation, event, or measurement, depending on the context. An event is a particular cognition by a focal cognizer about a given object state—“object” may be a system-within-the-world observed externally, or the cognizer’s environment. Two or more states, e.g., *u_1_, u_2_, …, u_n_,* of an object may be cognized as the same by a focal cognizer (*C*), e.g., *c_x_* → *c_y_* for those objects’ states. In this case, the same event occurs to the cognizer as the result of the cognition (observation) of these states; in other words, the cognizer cannot discriminate between these system states. Because the MO’s discrimination ability is perfect, each state of a CS corresponds uniquely to a cognition of the system by the MO. Therefore, for the MO, state and event are equivalent to each other. 

### 4.2. Cognition as Epistemic and Ontic Information

Epistemic information is knowing about an object. For a subject, knowing is the occurrence of events by observation of an object system or the subject’s environment. In the CS model, cognition is a state-change of a cognizer in relation to other cognizers; here, the state-change indicates that the former cognizer observes, or is informed of, the latter cognizers. Therefore, epistemic information is cognition—a state-change related to an observed system or the cognizer’s environment.

Ontic information is usually understood as pattern transmission occurring among entities within an observed system; “pattern” can be paraphrased as structure, configuration, or form. Any pattern that entities can form is represented as an interrelation among the states of the entities. Mathematically, a particular relation among the states of entities can be identified with a subset of the direct product of state-sets (or state spaces) of the entities (Appendix B). Therefore, given two cognizers (*A* and *B*), which are each composed of a plural number of sub-cognizers (i.e., cognizers at the next lower level of hierarchical organization of cognizers). Their state-changes (cognitions) involve state-changes of the sub-cognizers, and therefore, changes in their state-relation (pattern). Consider one-way pattern transmission from *A* to *B* within a given system where *B* cognizes *A*, leading to a particular state-change of *B* in relation to *A*’s state. Here, *B*’s state-change can be represented as a change in the internal state or state-pattern that component sub-cognizers form.

Notably, epistemic information can also be represented as pattern transmission in the CS model, when pattern transmission occurs from an object to an observing cognizer, a knower, which is composed of cognizers at the next lower level. Consider an observing cognizer *C_1_* composed of *k* cognizers *C_11_*, *C_12_*, ..., *C_1k_* at the lower level. When *C_1_* takes state *c_i_*, it is represented as a *k*-tuple of states of the lower-level cognizers, i.e., *c_1_* = (*c_1i_, c_2i_, …, c_ki_*). Therefore, a cognition for *C_1_* can be represented as (*c_1i_, c_2i_, …, c_ki_*) → (*c_1j_, c_2j_, …, c_kj_*) occurring in relation to the state of the environment harboring *m* cognizers, *e_i_* = (*e_1i_, e_2i_, …, e_mi_*). This representation of epistemic cognition shows that cognition involves changes in the internal state of a cognizer or the pattern that sub-cognizers form, as represented for ontic information described above. 

To conclude, epistemic information as knowing and ontic information as pattern transmission can be conceptually unified in terms of cognition. From the viewpoint of a cognizer as a knower (internal or external observer), cognition is event-occurrence by observation, generating changes in the internal state of the cognizer, in relation to an observed cognizer. From the viewpoint of a third cognizer (internal or external), cognitions between two groups of observed cognizers can generate pattern transmission from one group to another, which occurs within an observed system or within the environment for the third observer.

### 4.3. Discriminability and Selectivity of Cognition

Cognition determines one particular succeeding state of itself in relation to a focal object, such as an observed system for an external cognizer or the environment for an internal cognizer. This determination, performed by a focal cognizer with a particular property and represented by its motion function *f* (Section 3.2), involves two aspects of cognition: Discrimination and its ability, discriminability; and selection and its ability, selectivity. Discrimination aspect of cognition refers to a differential state change against different states of the observed system or the environment (its ability is called discriminability). The selection aspect of cognition refers to the selection of one particular succeeding state among the many possibilities in relation to others. The term “choice” is used for another meaning in the CS model, which means to choose a particular piece of information (data) for determining the succeeding state Nakajima [6]. The selection represents the *meaning* aspect of information by highlighting the relationship with other entities.

Accordingly, information has two aspects of cognition in the framework of the CS model. For example, a driver can discriminate colors of the signal light at an intersection, acting differently in response to them. This discriminability of cognition is the distinction aspect of information, which is focused by Shannon’s information theory [24]. Normal drivers stop, do not go, for the red signal. This is the selection (selectivity) aspect of information that cannot be manifested by the discrimination concept. Selectivity of cognition concerns the meaning of the red light. The states (colors) of a signal light relate a driver to the states of other cars. Based on a signal perceived, a succeeding state is selected (determined) by the driver, giving rise to a consequent relation with others. Living systems need to manage this meaning aspect of information, because they, as teleonomic systems, act to experience favorable events to maintain a particular relationship with their environments [6,25] (Section 6.3 in Reference [6]).

The term “choice” is used in the original form of Shannon’s theory [24], meaning the determination of one message among the many possible. Notably, this term represents the aspect of discrimination, not selection, in determination. This is because the theory does not address a particular functional relationship between a sender’s state and a sent message, i.e., the meaning content of each message. Weaver declares that “The concept of information applies not to the individual messages (as the concept of meaning would), but rather to the situation as a whole“ [24] (Chapter 2). A message sending–receiving process can be translated into the CS model as follows. Consider that a message–sender is an observed system, and a receiver is a cognizer observing the sender, external or internal to a given CS. Determination (choice) of one message by the sender means the determination of one particular sender state, being coded as a message among *n* possible ones, which is then sent. By receiving the message, a receiver then determines (changes from a previous state to) a particular state related to the sender state; in other words, the receiver can discriminate between *n* different states of the sender as an observed system. In this sending–receiving process, determinations (cognitions) performed by the sender and the receiver involve both discrimination and selection. Shannon’s theory focuses on the discrimination aspect of determination (cognition) by each of them. However, the theory does not deal with a particular relationship on which selectivity focuses between two states, before and after-in time determination for each, and that between the sender and receiver states, as the theory does not care about the contents of messages, focusing on only the number of messages. 

## 5. Probability 

### 5.1. Overview: Probability Concept in the CS Model

The above arguments provide a particular perspective for resolving the issue of the E-O duality by extending an ordinary cognition concept to a more general concept as a relational state change, applicable to either epistemic or ontic fields. This extension does not imply that higher-level cognitive processes in the brain can be simply reduced to cognition at a physical level, or that physical entities have mind-like properties. I review a probability theory using this extended concept of cognition and cognizer in the framework of the CS model, based on previous works on this subject [6,25,26,27] and develop it further for theorizing different concepts of entropy measure in a single theoretical framework of probability theory. 

Although the mathematical theory of probability has developed extensively since the axiomatization by Kolmogorov [28], a variety of interpretations of probability exist in science. The traditional interpretations of probability [1] include subjective (or epistemic) and objective interpretations. The former considers that probability means the subjective or epistemic degree of the certainty of the event occurring, whereas the latter considers that probability means the objective property of a system, which is represented in terms of relative frequencies of events in the system. However, these concepts are all probability for external observers or model builders. Nakajima [6,26,27] focused on the probabilities of events occurring to material entities within a material system, including the degree of certainty and relative frequency of events occurring to a material entity. This type of probability has not been addressed by the traditional probability interpretations. For example, consider the probability of a particular event that a bacterial cell experiences after a particular action, or the relative frequency of the event that the bacterium encounters predators without being eaten during a certain period of time. These probabilities can be considered from a viewpoint of an external observer whose observation does not affect the probabilities. However, a bacterium is a subject, like us humans, which also observes the external reality and acts in a particular way. Bacterial cognition or action affects the probabilities of events occurring to the bacterium. In this case, the probability is used for entities internal to an observed system, which is called “internal probability”, whether it is a degree of certainty or relative frequency in the long run.

I attempt to unify these different concepts, including some of the traditional interpretation relevant to science and internal probability, into a single framework in the CS model in which epistemic knowing and ontic observation/action are unified as “cognition” (Table 1). In this framework, the interpretation of probability varies according to the observer who experiences the event in question, and whether or not the probability depends on a particular observation. There are external and internal observers; the meta-observer is not included here, because it is not an observer that experiences events, instead of playing a role in the description and explanation of the probabilities of events occurring to cognizers in the world. In addition, the probability of an event can mean the degree of certainty of the event occurring under a particular observation (cognition) or the degree of how often the event occurs without reference to any particular observation (cognition). The former type is usually called an epistemic (or a subjective) concept of probability, whereas the latter the objective concept or the relative frequency concept. However, the former probability can be represented in terms of the relative frequency of the event occurring under a particular observation. For example, take a repeated coin-toss experiment: The relative frequency of coming up heads under a particular observation or data about the initial state of the experiment, which provides an epistemic degree of certainty of occurring heads in the future, if the initial condition of each toss is observed as the same. Therefore, this type is called as cognition-dependent probability, denoted P_cog_. The latter type of probability (usually called relative frequency) involves counting the number of a focal event occurring under overall observations, including different contents, to the total number of events occurring. Therefore, I call this type the overall-cognitions probability, denoted P_overall_. P_cog_ and P_overall_ concepts are each divided into sub-concepts depending on whether events are observed by external or internal observers. As summarized in Table 1, there are four types of probability.

Lastly, a certain type of subjective probability, called the degree of belief [1], should be mentioned here. Within the framework of the CS model, this type of probability is not identified as probability; instead, it is treated as a kind of mental state of a subject, related to the determination of a particular action among those available; such states are applicable only to human or related organisms with higher cognitive faculties. 

Let us consider an experiment of repeated coin-tosses for illustrating the concepts of P_overall_ and P_cog_ for external and internal observers. The MO describes a theoretical model for the behavior of the coin-toss system. This system is composed of a coin, a coin-tosser, a table, a person who observes this experiment, and others; these are all cognizers in the whole CS model. The person is an external observer (EO), who, as a cognizer, watches this experiment without affecting the coin-toss system (a part of the world). The coin-tosser is an internal observer (denoted as an IO), a cognizer within the coin-toss system, who interacts (inter-cognizes) with the coin. The coin, the table, and molecules in the air are all cognizers, which are subjects experiencing events. Therefore, these physical entities can also be called internal observers in the CS model, although they do not have enough internal memory to be called an “observer” in ordinary language. Here, let us focus on the two persons as observers: One internal (coin-tosser) and the other (watcher) external. 

### 5.2. Probability for the Meta-Observer (MO)

Events for the MO are identified in terms of “states” of the world or of its partial cognizers-systems. In other words, events occur in the world or partial systems. As represented previously (Section 3.2), the world, modeled as a cognizers system, is a state generator by which a succeeding state of the world is determined uniquely from a previous state. A cognizers system under consideration may consist of *n* cognizers; therefore, the states of the entire system can be represented in terms of the *n*-tuple of component cognizers’ states. Probabilities for the MO should not be differentiated into P_overall_ and P_cog_ because events are defined as subsets of system states. The MO can count the number of system states belonging to a given subset (**A_j_**) defined in terms of *n*-tuple states (e.g., a subset of the system states that the dice comes up heads), denoted #*A_j_*. It can also count the number of states of the entire set (**X**) of system states under consideration, denoted #*X*. The ratio of the former to the latter, i.e., #*A_j_*/#*X*, provides the probability. Here, #*A_j_* **= |A_j_|** and #*X* **= |X|** when states occur only once in the sequence, which is true in a deterministic system [26]. P_cog_ is given as the same manner as that producing P_overall_, except that a subset (**A_j_**) is defined in relation to a particular condition. The condition may be that states of **A_j_** have a particular relation with states occurring before in the time sequence.

This probability of an event is not a mere description of actual relative frequencies of events, but rather values actualized as a consequence of the system properties, *f_C_* and *f_E_*, in Section 3.2, evolving from a given initial state. In the CS model, the objective property of a system yields relative frequencies of events defined in terms of a subset of system states. This objective property of a system for yielding relative frequencies of events (defined in terms of a subset of system states) is similar to the propensity concepts of probability by Popper [29], although Popper denies the deterministic world.

### 5.3. Probability for External Observer (EO)

Events for an EO, including a measurement device, can be defined in terms of “cognition” by the EO, occurring in relation to particular states of an observed system. In other words, events are not something occurring in the observed system, instead of occurring to the EO in relation to the system. However, an event can refer to a particular state (or particular states) of the system because cognitions occur in relation to states of the system. Due to this relatedness, events occur in an EO as if they occur in the system (this aspect is explained in Section 7).

#### 5.3.1. External P_overall_

The P_overall_ measures the probabilities of events observed by an EO under various kinds of cognition (observation) by the EO (e.g., an observer of a coin-toss experiment from outside). The mechanism for determination is given similar to the MO, except that the EO’s discriminability is not perfect. Consider a partial system *S* with state-space **S** in the world *U*. A cognizer *C* as an EO, with state-space **C**, observes the system states by cognition. Consider a population of cognitions (*c_i_* → *c_j_*, including *i* = *j; c_i,_ c_j_* ∊ **C**) by observation that occurred during a certain period of time (note that any cognition can occur two or more times without violating determinism). The external P_overall_ of a focal event (cognition) is defined as the ratio of the number of focal events to the total number of events occurring in the population. 

*S* is usually composed by a plural number (say *n*) of cognizers. Therefore, the cognition *c_i_* → *c_j_* by an EO, including a measurement device (cognizer), may be represented as (*c_i1_, c_i2_,* …, *c_in_*) → (*c_j1_, c_j2_,* …, *c_jn_*), where component states correspond to states of the component cognizers in *S*. Here, an EO may focus on some of the system components. As stated previously, a cognizers system is a state generator by cognitions among cognizers. Therefore, events occurring to an EO are cognitions of these states generated by the observed system; in this sense, probabilities of the events are objective. Pattern formations or transfers occurring among cognizers can be described in terms of the probabilities (external P_overall_) of events referring to states of component cognizers. 

#### 5.3.2. External P_cog_

**Figure 3:**
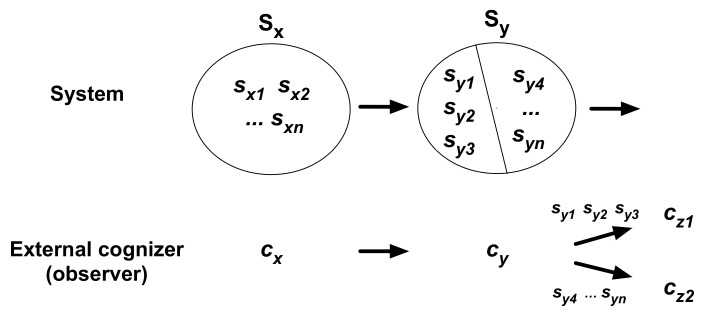
The degree of certainty of an event occurring to an external observer (cognizer) *C* with state-space **C**. *C* may include a measurement device (cognizer). *c_x_* → *c_y_* indicates an observational cognition of the system, and *c_y_* → *c_z1_* or *c_z2_* indicates resultant cognitions of the external cognizer *C*. *s_xi_* → *s_yi_* (1 ≤ *i* ≤ *n*) represents a cognition (state-change) of the entire system *S* with state-space **S** observed by the external observer. Arrows indicate state-changes, which may include intermediate states between a given state and the next state.

The external P_cog_ measures the probabilities of events observed by an EO under a particular cognition (observation) by the EO (e.g., an observer of a coin-toss experiment from outside). The mechanism for determination is illustrated in Figure 3. State changes of an EO and an observed system are indicated with arrows, in which intermediate states may exist between the states shown. The EO in a given state (*c_x_* ∊ **C**) cannot discriminate between different states of the observed system *s_x1_, s_x2_, …, s_xn_* (∊ **S_x_** ⊂ **S**), i.e., the EO cognizes them as the same, changing to *c_y_* (∊ **C**). This cognition *c_x_* → *c_y_* is an observation (cognition) of the system (e.g., “a coin was tossed in such and such a way”). Corresponding to this observational cognition, *n* resultant states s*_y1_, s_y2_, …, s_yn_* (∊ **S_y_** ⊂ **S**) occur—there are *n* resultant states if the system changes states in a one-to-one mapping under the assumption that the system is effectively isolated from the system’s environment. If the EO cognizes either of the three states, *s_y1_, s_y2_, s_y3_,* as the same resultant event *c_y_* → *c_z1_* (e.g., heads), the P_cog_ is given as 3/*n* (Figure 3). This is the external probability (P_cog_) of an event (*c_y_* → *c_z1_*) occurring under the conditional cognition or observation (*c_x_* → *c_y_*). The state changes, in the above illustration, are extracted from a continuous state sequence, such as: …, (*c_x_*, *s_x1_*), (*c_y_*, *s_y1_*), (*c_z1_*, ∙), …, (*c_x_*, *s_x4_*), (*c_y_*, *s_y4_*), (*c_z2_*, ∙), ….

Observational cognition is not necessarily a prediction of a resultant event, which may be a visual perception or a hearing of sounds. If *c_x_* → *c_y_* is a perception whose semantic content is that “the coin will come up heads”, and if the resultant observation is always “tails”, the probability of tails under this predictive perception (*c_x_* → *c_y_*) is 1; i.e., the result under the prediction is completely certain. The semantic contents of a prediction are a matter of code that links a cognition and a relation between a subject and an observed system, as illustrated in Section 4.3, using driver’s selectivity to signal-light colors.

### 5.4. Probability for Internal Observer (IO)

Events for an IO can be defined in terms of cognition by the IO, occurring in relation to particular states of the environment. In other words, events are not something occurring in the environment, instead of occurring to the IO in relation to the environment. However, an event can refer to a particular state (or particular states) of the environment because cognitions occur in relation to states of the environment. Due to this relatedness, events occur to an IO as if they occur in the environment (this aspect is explained in Section 7). IOs may include a measurement device. Traditional probability concepts have not focused on this type of probability. This concept was formalized by Nakajima [26,27] and named internal probability, which includes internal P_overall_ and P_cog_ types. The internal probability should play an essential role in explaining living processes because living systems are internal observers or players within an ecosystem that cope with their environments to survive and reproduce.

#### 5.4.1. Internal P_overall_

The internal P_overall_ measures the probability of events occurring to (experienced by) an IO under various kinds of cognition (action) by the cognizer (e.g., a coin-tosser). The mechanism for determination is given similarly to the external P_overall_, except that IOs interact with cognizers of the system to which they belong. Consider an observed system *S* with state-space **S** in the world *U*. A cognizer *C*, as an IO with state-space **C**, observes the environment *E* with state-space **E** by cognition; cognition is not restricted to a change in internal states, which can include changes in physical states, such as position, velocity, and others. Consider a population of cognitions (*c_i_* → *c_j_*, including *i* = *j; c_i,_ c_j_* ∊ **C**) by its observation, which occurred during a certain period of time. The internal P_overall_ of the event (cognition) in focus is defined as the ratio of the number of events to the total number of events occurring in the population. The major difference of this internal P_overall_ from the external P_overall_ is that the environment interacts with the IO. 

#### 5.4.2. Internal P_cog_

**Figure 4:**
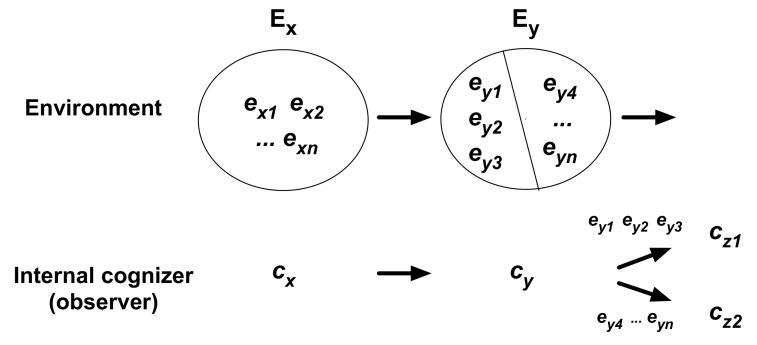
Degree of certainty of events (internal P_cog_) occurring to a focal internal observer (cognizer) *C* with state-space **C**. *c_x_* → *c_y_* indicates an observational cognition of the environment and *c_y_* → *c_z1_* or *c_z2_* indicates resultant cognitions of the internal cognizer *C*. *e_xi_* → *e_yi_* (1 ≤ *i* ≤ *n*) represents a cognition of the environment, where *e_yi_* = *f_E_* (*c_x_, e_xi_*). Arrows indicate state changes, which may include intermediate states between a given state and the next state.

The internal P_cog_ measures the probability of events occurring to (experienced by) an IO under a particular cognition (action) by the cognizer (e.g., a coin-tosser). The mechanism for determination is illustrated in Figure 4. The determination is similar to external P_cog_ but the important difference is that a focal cognizer interacts with the environment. State-changes of an IO (focal cognizer) and the environment are indicated with arrows, in which intermediate states (not shown in the figure) may exist between the states. The IO in a given state (*c_x_* ∊ **C**) cannot discriminate between different states of the environment *e_x1_, e_x2_, …, e_xn_* (∊ **E*_x_*** ⊂ **E**). In other words, the IO cognizes them as the same, changing to *c_y_* (∊ **C**). This cognition *c_x_* → *c_y_* is an action (cognition) of the environment. Corresponding to this action, *n* resultant states *e_y1_, e_y2_, …, e_yn_* (∊ **E*_y_*** ⊂ **E**) occur; there are *n* resultant states if the system changes states in a one-to-one mapping under the assumption that the system is effectively isolated from the system’s environment. If the IO cognizes either of the three states, *e_y1_, e_y2_, e_y3_,* as the same resultant event *c_y_* → *c_z1_* (e.g., heads), the P_cog_ is given as 3/*n* (Figure 4). This is the probability (internal P_cog_) of an event (*c_y_* → *c_z1_*) occurring under the conditional cognition or action (*c_x_* → *c_y_*). The state changes in the above illustration are extracted from a continuous state sequence, such as: …, (*c_x_*, *e_x1_*), (*c_y_*, *e_y1_*), (*c_z1_*, ∙), …, (*c_x_*, *e_x4_*), (*c_y_*, *e_y4_*), (*c_z2_*, ∙), …

In a coin-toss experiment, each cognition by the coin-tosser is a process of sensing-and-acting involving the sensors, the brain, and effectors. A coin-tosser experiences a particular probability (P_cog_) distribution of events (heads and tails) corresponding to a particular kind of action of tossing (i.e., cognition *c_x_* → *c_y_*). Consider a skillful coin-tosser. They could experience a biased probability (internal P_cog_) of heads or tails by manipulating the coin-toss [30]. The coin is also a cognizer that cognizes the coin-tosser and changes its state. A biased coin may cognize the coin-tosser’s hand and the table surface differently compared with a normal coin. 

As stated previously, internal observers in this framework are not restricted to cognizers equipped with a certain amount of memory and information processing ability. Even molecules can act non-randomly due to their electromagnetic properties in water, for example. Therefore, their chemical properties can affect encounter probabilities, in terms of internal P_cog_ and P_overall_, with other molecules, and can affect chemical-reaction rates within a living cell [31,32]. 

### 5.5. Relationship between P_overall_ and P_cog_

Let us address the next question as to the underlying relationship between P_overall_ and P_cog_, external or internal, which remains unclear. As demonstrated in Figure 3 and Figure 4, the probability (P_cog_) of a resultant cognition (event) (e.g., *c_y_* → *c_z1_*) under a particular cognition (*c_x_* → *c_y_*) is a conditional probability determined for the observational cognition; this probability is obtained by the ratio of the number of a focal resultant cognition (3 occurrences of *c_y_* → *c_z1_*) to the total number of resultant cognitions (3 occurrences of *c_y_* → *c_z1_* plus [*n* – 3] occurrences of *c_y_* → *c_z2_*). 

The same event, such as *c_y_* → *c_z1_*, can occur under other observational cognitions for conditions. Using mathematical expression of conditional probability, the P_cog_ of event *A_i_* (e.g., *c_y_* → *c_z1_*), occurring under cognition *B_i_* (*c_x_* → *c_y_*) earlier in time, is expressed as P(*A_j_*|*B_i_*) (= 3/*n* in Figure 4). P(*B_i_*) is the P_overall_ of *B_i_*. Therefore, $\sum_{i=1}^{N}P\left(A_{j}|B_{i}\right)P\left(B_{i}\right)=P\left(A_{j}\right)$. This equation indicates the relationship between the P_cog_ of event *A_j_* under observational cognition *B_i_*, and the P_overall_ of event *A_j_* (Appendix C for a general representation). In other words, the P_overall_ of event *A_j_*, P(*A_j_*), is obtained by summing P(*A_j_*|*B_i_*)P(*B_i_*) for various cognitions *B_i_* as conditions for occurrence of *A_j_* in a state sequence.

Remember that, in repeated coin-tosses, the P_cog_ of heads is 1 or 0 under observations by the Laplace’s demon [33], to which no uncertainty exists in the prediction derived from their observation (cognition). However, the P_overall_ of heads is nearly one-half, even to this omniscient entity. This relationship between the P_cog_ and P_overall_ of events can be explained using the above formulation: P(*A_j_*|*B_i_*) = 0 or 1 according to the demon’s observation, *B_i_*. *B_i_* includes two kinds of observations: “Such and such an initial state was observed; therefore, it will come up heads” (denoted *B_H_*) and “such and such an initial state was observed; therefore, it will come up tails” (denoted *B_T_*). Assume that the initial states of the repeated coin-toss systems are random, i.e., P(*B_H_*) ≈ P(*B_T_*) ≈ 1/2, where P(*B_i_*) is P_overall_ of observational events of the initial states of the system—the trials of coin-toss can also be considered to be connected into a single, continuous state-transition of system. Let event “heads” be denoted as *A_H_*. Therefore, P(*A_H_*|*B_H_*)P(*B_H_*) ≈ 1 × 1/2, and P(*A_H_*|*B_T_*)P(*B_T_*) ≈ 0 × 1/2, for the demon. Therefore, P(*A_H_*|*B_H_*)P(*B_H_*) + P(*A_H_*|*B_T_*)P(*B_T_*) = P(*A_H_*) ≈ 1/2, where P(*A_H_*|*B_H_*) is the P_cog_ of heads (*A_H_*) under observation *B_H_*, P(*A_H_*|*B_T_*) is the P_cog_ of heads under observation *B_T_*, and P(*A_H_*) is the P_overall_ of heads. This relationship between the P_overall_ and the P_cog_ is true for the cases of EOs and IOs, for which P(*A_H_*|*B_i_*) varies between 0 and 1 according to their discriminability.

### 5.6. What Determines P(B_i_)?

P(*A_j_*|*B_i_*) discussed in the above section can include external and internal P_cog_, and the mechanisms for determination are elucidated in Figure 3 and Figure 4, respectively. What about P(*B_i_*)? Let us discuss what determines P(*B_i_*) for the case of EOs who observe experiments. Experiments are partial systems constructed by a scientist, an external cognizer. A plural number of replicate systems can be constructed and run in parallel along the same time course or sequentially in time. A coin-toss experiment can be understood as a simple model for experimental systems in science. A coin-tosser can be replaced with a coin-tossing robot if they want to remove the complex human factor from an experimental system. Consider a relationship between two events, i.e., an observation at the beginning of coin-tossing, and an observation of the result, heads or tails. The first observation is of the initial state of the coin-toss experiments. When coin-toss experiments are repeated, in parallel or sequentially, the results show a nearly 50:50 ratio of heads:tails. This result indicates that the initial states (conditions) were distributed almost evenly for those resulting in heads and those resulting in tails: P(*B_H_*) ≈ P(*B_T_*) ≈ 1/2, where *B_H_* and *B_T_* are subsets of observational events (initial-state events) resulting in heads and tails, respectively, as above. Here, an initial state is realized by an observation (cognition) for an EO. P(*B_i_*) indicates a probability distribution of observations, *B_i_*, about initial states for particular kinds of experiments which are repeated. 

What then determines P(*B_i_*)? This probability distribution does not depend on a particular observation; in other words, it is P_overall_ distribution and is unrelated to knowledge. If determinists seek to explain the probability distribution in terms of system states earlier in time based on causality, then they would be led to an infinite regress, as discussed by Landé [34] and Popper [29]. In the CS model, the answer is that P(*B_i_*) is determined by the entire CS (i.e., the world), which includes an external cognizer observing a partial system, or an internal cognizer observing its environment (Figure 1). This answer is also true for the cases of IOs because the relative frequency of observational events (*B_i_*) occurring to an IO is determined by the entire CS.

## 6. Entropy and the Amount of Information

### 6.1. Overview

Probability focuses on measuring the degree of occurrence of a particular event, which does not address the diversity of events. Entropy measures the diversity of events with their probability distribution. Entropy plays a powerful role in measuring the uncertainty of events occurring as the epistemic aspect of diversity, or in measuring the (dis)organization or (dis)order of an observed system or the environment as the ontic aspect. In the previous section, four types of probability were presented depending on whether a cognizer (observer) is inside a system or outside and on conditions for determination (Table 1). Therefore, four types of entropy, *H* = $\sum_{i}P_{i}\log_{2}P_{i}{}^{-1}$ using the four types of probability, can be derived accordingly. They are measures for the uncertainty of events or for the disorder of a system, with each for IOs or EOs. In addition, four types of the amount of information are discussed as a measure of uncertainty reduction and disorder reduction, respectively, for IOs or EOs. 

### 6.2. External Entropy (H_cog_) and the Amount of Information (I_cog_)

Entropy for external observers (cognizers) is called the external entropy, which includes H_cog_ and H_overall_. H_cog_ is obtained from a probability (P_cog_) distribution of events experienced (observed) by an external cognizer (observer) under a particular cognition (observation) by the cognizer, denoted external H_cog_. According to Shannon’s mathematical theory of information (or communication), the amount of information is the amount of entropy (*H* = $\sum_{i}P_{i}\log_{2}P_{i}{}^{-1}$) reduction by observation or receiving a message. Therefore, the external H_cog_ is a typical case of entropy described by Shannon’s information theory. In other words, this entropy measures the uncertainty of events occurring under a particular kind of observation by an EO. Here, a particular kind of observation is identified by a particular kind of state change of the EO in relation to states of an observed system. 

Information, i.e., cognition, by an external cognizer affects probabilities of events occurring to the cognizer under the information, hence affecting the entropy value. The amount of information measures the degree of a difference in external H_cog_ that is generated by a difference in information. Information for EOs is knowing (obtaining data) by observation. Therefore, a difference in data may generate a difference in the entropy (H_cog_) value about an observed system. The difference in H_cog_ is the amount of information (I_cog_) for an EO, denoted external I_cog_. External I_cog_ is identical to Shannon’s amount of information in its ordinary sense. Here, information indicates “distinction” or “choice” undergone by receiving a message, where a message-sender corresponds to an observed system, and a receiver to an EO. In other words, information as distinction reduces uncertainty about an object. 

Remember that information defined as cognition has two aspects of cognition: Discrimination and selection (Section 4.3). The main reason why Shannon’s theory addresses the amount of information, and not the meaning of information, is because the theory implicitly defines information as the distinction (discrimination) and ignores its selection aspect. For example, the H_cog_ value is the same in the cases of the 80:20 ratio and the 20:80 ratio of heads to tails occurring under a particular cognition by an EO of a coin-toss experiment. However, the two cases might have different meanings and worth for the EO if heads and tails can have different importance for the EO. The aspect of information relating to meaning or worth is important when the EOs considered are living systems because they need to maintain a particular relation with their environments. 

### 6.3. External Entropy (H_overall_) and the Amount of Information (I_overall_)

H_overall_ is obtained from a probability (P_overall_) distribution of events experienced (observed) by an external cognizer (observer) under various kinds of cognition (action) by the cognizer; denoted external H_overall_. As stated previously, P_overall_ is usually called relative frequency. Distribution of external P_overall_ values can represent the degree of disorder or disorganization, which can be measured by H_overall_, denoted as external H_overall_, using Shannon’s H where external P_overall_ is used for *P*. External H_overall_ is the entropy independent of a particular observation (information, cognition). Disorder or disorganization becomes an objective description by defining it formally, although it sounds subjective when used in isolation from a formalism.

Consider a system observed ay an EO. The system is composed of cognizers *C_1_, C_2_, C_3_*, …, *C_n_*. The EO observes their state-changes and obtains a set of events that refer to their states (Section 5.3). From the relative frequencies of these event occurrences, the EO can obtain the probabilities (external P_overall_) of events (cognitions) referring to the states of components (cognizers) in the observed system during a particular period of time. When the distribution of probabilities (P_overall_) of events about these components is more uniform, producing a higher external H_overall_ value, the system has visited a wider area of its state space.

Let us consider the amount of information for external H_overall_. Recall the example of a repeated coin-toss experiment. The external P_cog_ of coming up heads is between 0 and 1, which depends on the ability to discriminate the coin-toss system. If an EO has a higher discriminability, the P_cog_ of heads (or tails) approaches 1 or 0 for an observation; therefore, external H_cog_ approaches near zero. However, the external P_overall_ of heads (or tails) remains near ½ for any observer, the demon, or humans, which is an objective property of the system side to yield such a frequency of coming up heads (or tails). External H_overall_ may not be affected by a difference in the observer’s discrimination ability under the assumption that the observers under consideration can identify, at least, outcome events distinctly (e.g., heads and tails). Under this assumption, external H_overall_ values obtained by different EOs are the same, and therefore the amount of information (i.e., external I_overall_) obtained by altering the EO is zero. In this sense, external P_overall_ and P_overall_-based entropy (i.e., external H_overall_) appear to be objective descriptions of an observed system. 

The external H_overall_ changes if the functions (properties) of cognition (*f_C_, f_E_* in Section 3.2) are altered. For example, compare a coin-toss experiment using a biased coin and using a normal coin. In this case, their external H_overall_ values should be different. Therefore, this difference (i.e., external I_overall_) can measure the amount of a certain type of ontic information, which is the production of a pattern or order by interactions among entities. In a famous thought experiment proposed by Maxwell [35], an imaginary being, later called Maxwell’s demon, generates a non-uniform distribution of molecules in a vessel divided into two portions by a wall with a small hole. The demon affects the related state-changes of molecules by opening or closing the hole. If the demon is interpreted as an imaginary trick to understand the effect of an alteration of the physical properties of molecules concerning how they act (move), this imaginary experiment would suggest that *discriminative* and *selective* cognitions (i.e., actions or movements) by each system component can generate a particular pattern measured in terms of external H_overall_. The amount of information, in the sense of forming a pattern (external I_overall_), can be measured by a difference in the external H_overall_ values between two systems, as above. Recently, a new approach using topological techniques (TDA) is being developed to process very large data sets, known as big data [36,37]. This approach is based on information (data) as patterns or a form of data-point clouds sampled (observed) from an observed system for an EO (data analyst). The topological forms may be related to external H_overall_. 

### 6.4. Internal Entropy (H_cog_) and the Amount of Information (I_cog_)

Entropy for internal observers (cognizers) is called the internal entropy (Nakajima [6]), which includes H_cog_ and H_overall_, each obtained by using the equation of Shannon’s H. H_cog_ is obtained from a probability (P_cog_) distribution of events experienced by an internal cognizer under a particular cognition (action) by the cognizer, denoted internal H_cog_, which is represented in Figure 4. Here, events are identified by cognitions by an IO, as represented by *c_x_* → *c_y_* and *c_y_* → *c_z1_ or c_z2_* in Figure 4, which are not identified by an EO, such as a scientist. For example, an organism experiences various kinds of events as a result of a particular action with a certain probability distribution of events.

Information, i.e., cognition, by an internal cognizer affects internal P_cog_, and hence internal H_cog_. The amount of information measures the degree of a difference in entropy generated by a difference in an information process. Consider the following case in Figure 4. Suppose that the cognition *c_x_* → *c_y_* becomes more discriminative: For example, *c_x_* → *c_y_* for *e_x1_, e_x2_*, and *e_x3_*; and *c_x_* → *c_y_’* for other states of *E*. In this case, under *c_x_* → *c_y_*, the cognizer experiences the consequent event *c_y_* → *c_z1_* with a probability of 1 (P_cog_ = 1, H_cog_ = 0). As such, a probability distribution following a given cognition is altered by alteration of that cognition. The difference in the first H_cog_ minus the second H_cog_ can represent the amount of information of the second cognition relative to the first (i.e., internal I_cog_). The internal I_cog_ measures the amount of uncertainty reduction by the action for the material entity. It is a ubiquitous property that living systems possess relatively high abilities to discriminate between different states of environments and act selectively (appropriately). The internal I_cog_ can measure their properties of cognition relative to those of their ancestors. 

It is sometimes stated that environments are uncertain for living systems. This may be true for an EO because the uncertainty of system behavior reflects mainly system properties involving uncertain actions from the outside (Figure 1). However, it is not the environment that is uncertain for IOs. The uncertainty of events occurring to IOs is not determined only by the environment side, but by interactions between a focal subject and the environment. Information (cognition) does not occur in one way, but in both ways among cognizers. Depending on their discriminative and selective cognitions, the environments for IOs, such as living systems, are not necessarily uncertain in terms of internal H_cog_. These interactions are not effectively modeled in Shannon’s framework of communication systems.

### 6.5. Internal Entropy (H_overall_) and the Amount of Information (I_overall_)

H_overall_ for an IO is obtained from a probability (internal P_overall_) distribution of events experienced by an internal cognizer under various kinds of cognition (actions) by the cognizer, denoted internal H_overall_. For example, an organism experiences various kind of event with a particular probability distribution during the lifetime. Again, events are identified by cognitions by an IO, not by an EO. The relationship between H_overall_ and H_cog_ is understood from the relationship between P_overall_ and P_cog_ (Section 5.5 and Appendix C). In Maxwell’s thought experiment discussed previously for external H_overall_ and I_overall_, a pattern formation (i.e., becoming non-uniform distribution) of molecules is viewed from an EO. Now, we can understand another implication of this experiment by shifting the observer from an EO to an IO—a probability distribution of events viewed from the demon as an internal cognizer. Suppose that molecules in a vessel do not have discriminative and selective properties to generate a non-uniform distribution themselves. However, through discriminative and selective actions by the demon, the demon experiences events of a non-uniform molecular distribution (internal H_overall_). This non-uniform distribution can also be observed for any EO in terms of external H_overall_. The important point of this internal view is that the probability distribution depends on the IO, unlike in the case of EOs, because it is a consequence of interactions (intercognitions) between the IO and other components in the system. 

Similarly to internal P_cog_, an alteration in cognitive properties affects internal H_overall_. Accordingly, a difference (i.e., the internal I_overall_) between internal H_overall_ for a referential original cognizer and that for a cognizer with a new cognitive property, can measure the amount of information of the alteration of cognition property relative to the original property (i.e., internal I_overall_). This measures the effect of an alteration of cognitive properties on internal H_overall_. 

### 6.6. H_cog_ and H_overall_ for Living Systems

The internal H_cog_ and H_overall_ play an essential role in understanding living systems because they focus on the probabilities of events experienced by internal cognizers in relation to their actions and the properties of how they act in response to their environments. A question then arises as to whether there is any tendency or principle about the relationship between biological evolution and H_cog_ or H_overall_.

Internal H_cog_ measures the uncertainty of events followed by a particular cognition or action. Therefore, it is conceivable that lower values of internal H_cog_ are better for a living system, as long as favorable events for survival and/or reproduction occur at higher probabilities than less favorable events. However, as widely known in evolutionary ecology [38,39], there may be a trade-off between organism phenotypic traits that increase survivability by discriminative and appropriately selective actions using developed sense-organs and brains (e.g., vertebrates), and traits that increase reproductivity by yielding more offspring (e.g., invertebrates). Due to this trade-off, living systems with a higher internal H_cog_ than alternative types can evolve in a certain environment, where traits reducing the internal H_cog_ confer a relatively high cost on reproduction due to physiological or developmental constrains. Under such a condition, genetic types yielding more offspring or eggs can achieve a higher lifetime net reproductive output (i.e., a higher fitness) despite low survivability. Therefore, the internal H_cog_ for living systems tends to decrease through natural selection; however, in general, it may not necessarily decrease due to the trade-off between traits increasing survivability and reproductivity.

The internal H_overall_ is also related to living systems and their evolution because they require an appropriate probability distribution of events in their lifetime to maintain their internal order (i.e., survival) and reproduce. Some events, such as encounters with resources or prey organisms in a lifetime, can increase both survival and reproductivity; some events, such as encounters with predators, can decrease survivability, and others such as encounters with mating partners, can increase reproductivity. It is conceivable that the best lifetime probability distribution, in terms of the relative frequencies of events experienced by a living system (i.e., the internal H_overall_), exists depending on its biotic and abiotic environment. Therefore, hypothetically, the internal H_overall_ for living systems neither tends to decrease nor increase but converges toward a particular range of values in evolution, depending on their survival/reproductive strategies and niches in ecosystems. Lastly, the concept of the probability distribution in the internal H_overall_ ignores the order of occurrence of events in a lifetime, which is important for determining survivability and reproductivity for living systems. A good theoretical measure is required for estimating an overall (lifetime) survival-and-reproductive success (i.e., fitness) of living systems in the CS model, which should be developed in the future.

## 7. An Internalist Model: Realization by Inverse Causality

### 7.1. Overview

**Figure 5:**
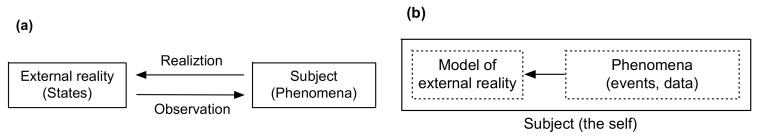
(**a**) The circularity of entailment between epistemic (“phenomena”) and ontic (“external reality”) fields, i.e., the E-O circularity. (**b**) The internalist model representing a possible way for the subject to construct a model of the external reality within based on phenomena or data.

I address the issue of E-O circularity, the interdependence between observation and the external reality. Observations of the external reality produce phenomena (or events) in a subject, whereas the external reality is realized (constituted) from phenomena (Figure 5a). For a subject, the occurrence of phenomena or data is not necessarily “observation” of something, because nothing might exist outside the subject, such as in dreams and hallucinations. Derivation of something external from phenomena is called “realization”. From an internalist point of view, I aim to develop a conservative model without assuming the external reality and the world (*E* and *U* in the CS model, respectively; Section 3), in order to understand how a given subject can constitute an internal model of the external reality based on a given sequence of phenomena or data (Figure 5b). To this aim, I first review an internalist model of realization by inverse causality (denoted the inverse-causality model) developed by Nakajima [6,23], then attempt to link this model to the CS model, an externalist model, toward a comprehensive information theory. 

The terms “phenomena”, “data”, and “events” are used interchangeably, which are represented as *m_i_* → *m_j_* in the model. The term “internal model” is used to mean a model of the external reality constituted within a subject (cognizer) with memory and data (percepts) processing capabilities, like living systems, in the world. The term “internalist model” is used to mean a model based on a stance of internalism as a fundamental stance for the mind–world relationship, which expounds that phenomena occurring to a subject, or mind activities, depend only on the subject [40,41].

### 7.2. Realization by Inverse Causality

As a sequence of data (percepts or sense data; Appendix D for sense data), which are represented as meaningless symbols, it is the primary sequence of data, *M* with the set **M** of symbols. Foreign symbols, which do not belong to the first sequence, are derived from the first sequence by a principle (or algorithm), called the inverse causality, originally developed by Nakajima [23], which is the contrapositive of the principle of unique-successor.

The unique-successor principle stipulates that “Every element in the temporal sequence has a unique successor”. Inverse causality is defined as: “If a given perceptual sequence satisfies the unique successor principle, no operation is required. If not, new foreign elements are introduced into the sequence in order that every perceptual element is a unique successor of an immediately previous element of the new sequence”. The inverse causality (if *F(a) ≠ F(b),* then *a ≠ b*) is the contrapositive of the unique-successor principle (if *a = b,* then *F(a) = F(b)*). Note that this principle does not exclude the case where different elements are followed by the same element; that is, *F(a) = F(b)* when *a* ≠ b.

Consider the simple example of a sequence of data occurring in succession, *M*:

*m_0_, m_1_, m_2_, …, m_0_, m_1_, m_3_, …,* (1)

where *m_i_ = m_j_*, if and only if *i = j*; **M** = {*m_0_*, *m_1_, m_2_, m_3_, …*}. This sequence does not fulfill the unique-successor principle because the first *m_1_* is followed by *m_2_*, and the second *m_1_* by *m_3_*. Therefore, through an operation of inverse causality on *M* (ICM)*,* the first and second *m_1_* are differentiated by introducing the foreign symbols e_0_* to the first *m_1_*, and e_1_* to the second one; here, derived symbols are marked with an asterisk (*) to distinguish them from symbols used for states of the environment in the CS model (Section 3). e_i_* is not a state of the external reality, but a state of the subject that refers to something external. Therefore, the sequence now becomes

m_0_, (m_1_, e_0_*), m_2_, …, m_0_, (m_1_, e_1_*), m_3_, …, (2)

The consequent sequences are generated downstream of *M*, i.e., within the subject. 

From a viewpoint of the meta-observer in the CS model, the operation of inverse causality on *M* can be interpreted as a measurement process detecting the external reality with state set E_0_* = {*e_0_, e_1_*}. Measurement is a causative discrimination process by a measurer between different states of an external reality [42,43]. Accordingly, the perceptual changes occurring at *m_1_*, such as (m_1_, e_0_*) → *m_2_*; (m_1_, e_1_*) → *m_3_*, can be a process of cognitive discrimination about something by the subject. Using the state concept, the differences to be discriminated are differences in the state of something. 

Noticeably, Sequence (2) does not fulfill the unique-successor principle at the whole level because it contains a non-unique determination: *m_0_* → (m_1_, e_0_*), or *m_0_* → (m_1_, e_1_*), although it fulfills the principle at *M* level. Therefore, inverse causality is, again, operated for Sequence (2) at the whole level (denoted as ICW). To fulfill the unique successor principle, new foreign symbols, e_2_* and e_3_*, must be introduced to the first and second *m_0_*. Then, the following sequence is obtained:

(m_0_, e_2_*), (m_1_, e_0_*), m_2_, …, (m_0_, e_3_*), (m_1_, e_1_*), m_3_, …, . (3)

By operation of ICW, the symbols referring to the external reality E_1_* are constituted with state set E_1_* = {e_0_*, e_1_*, e_2_*, e_3_*}. The ICW process constitutes symbols for an undetectable reality (E_1_*) mediated through an ICM-constituted reality (E_0_*) within the subject. The derivation of symbols by ICM is a minimum realization process without recourse to the semantic contents of individual percepts, whereas the derivation by ICW is more progressive, which is mediated through a directly-derived (ICM-constituted) reality. The ICW-based measurement includes device-mediated measurements, which are widespread in natural sciences, including physics, chemistry, and biology. 

Without ICW, the first and second *m_0_* → *m_1_* in Sequence (1) can produce no information in terms of Shannon’s information or Bateson’s information as “any difference that makes a difference” [44]. The ICW process explicitly represents the it-from-bit type of information. According to Wheeler [16], an elementary quantum phenomenon is “the elementary device-intermediated act of posing a yes-no physical question and eliciting an answer”. He calls this device-intermediated realization of “it” as “it from bit”: “Every **it**—every particle, every field of force, even the spacetime continuum itself— derives its function, its meaning, its very existence entirely—even if in some contexts indirectly—from the apparatus-elicited answer to yes or no questions, binary choices, **bits**” (Bold face in the original text). A subject, such as an experimenter, obtains a bit about a physical object (e.g., the detection/non-detection of a photon) mediated by the device. This device-mediated derivation of an external reality is equivalent to ICW. This poses a question as to whether our universe is deterministic or indeterministic (Appendix E). 

The ICM process directly measures the external reality in different states. What then does the ICW process measure? The ICW process operates on perceptual transitions, *m_0_* → *m_1_* → *m_2_* or *m_3_*. This pattern of transitions has the same structure of internal probability (Section 5.4.2). From a determinist viewpoint, chance arises from ignorance about the object [33]. In this sense, the ICW process measures something hidden that cannot be directly observed, only mediated through probabilistic events.

As an internalist model, the inverse-causality model starts with a temporal sequence of primary data, which do not fulfill the principle of causality, and nothing is assumed that entails or causes the data. ICM/W operate on a memorized sequence of the data (or percepts) occurring in succession (Sequence (1)) to produce a sequence of derived or secondary data that fulfill the causal principle (Sequence (3)). Through this process, the derived sequence contains something that entails or causes the primary data. The ICM/W processes are impossible for a subject without any capacity for memorizing a temporal sequence of data, which exists at every moment of now. Although data sequences in memory are timeless in the sense that data synchronically form a (fragment of) sequence, their sequences contain a temporal order or structure. ICM/W processing proceeds in the direction of time, i.e., from the present to the future. However, it proceeds data-in-sequence in the opposite direction of their temporal relations, i.e., from data occurring later to earlier. Here, it is important to distinguish the logic of the principle of causality and of its contrapositive, the inverse causality, from a material process that obeys the principle. Bateson [44] points out that the *if, then* of causality and that of logic are different; the former contains the time, whereas the latter is timeless. 

Lastly, let us focus on the diversity of internal models of the external world (the environment). How does the inverse-causality model relate to this diversity? ICM/W-constituted reality (E_0_* and E_1_*) in the above description is a set of symbols referring to the whole external reality that has not been differentiated into individual objects, such as oxygen and nitrogen molecules or two balls on a table, for example. The differentiation of a constituted reality requires ceteris-paribus ICM/W measurements of a focal object by assuming states of the remainder of the constituted reality are the same. One possible source for generating a diversity of internal models among subjects arises from differences in the dataset that each subject has. Another source can arise from differences in the choice of data used for ICM/W processing from the dataset, depend on different degrees of importance of data for subjects. This variation in the internal model about the external reality can explain the diversity of internal models in the living systems, which may correspond to what Uexküll [19,20] called “umwelt”. All the data that can be obtained are restricted by sensors (molecules or organs) for organisms, and by measurement devices for scientists. Data are chosen from the entire dataset obtained by a subject, and the reality is divided into plural objects as an internal model, depending on the strategy to survive/reproduce for organisms. Similarly, in science, a variety of world views (models) have been proposed depending on the group of scientists in various fields, and also on differences in research interest (curiosity) and abductive reasoning for scientists.

### 7.3. Cognizer Equipped with an Internal Model

The above processing of symbols by ICM/W requires a processor and the memory of data sequences, which are not explicitly described in the inverse-causality model. If a subject does not have any processor and memory for data, the subject exists in the form of a single phenomenon (*m_i_*) occurring now, with each being replaced by another occurring another now. With additional assumptions explicit in the CS model, the subject *M* can be modeled as a certain type of cognizer, which has a certain amount of memory and an information processing capability, like living systems and robots. In the following, the ICM process is incorporated into the CS model (ICW is not the focus here). 

Consider a system and an external observer as represented in Section 5.1 (Figure 3). The observer consists of two partial cognizers, sensor, and memory cognizers, which are represented as (sensor state, memory state). The sensor can change its state from *0* (as a basal state) to another, such as *0* → *1*, *0* → *2*, …, *0* → *n*, as measuring cognitions through which it can discriminate between *n* differences about the system. Arabic numbers are used for simplicity that does not necessarily represent quantity, but they can. It is assumed that the sensor returns to the basal state *0* after measurement. This property is similar to neural cells. In addition, the memory can take states *μ_0_*, *μ_1_*, …, *μ_n_*.

**Figure 6:**
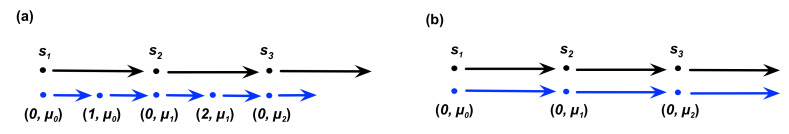
Observation (blue arrows) of an object system *S* by an external observer using inverse causality processing (ICM). (**a**) State transition of an object system *S*, *s_1_* → *s_2_* → *s_3_*, …, *s_n_*; and that of its observer, (*0, µ_0_*) → (*1, µ_0_*) → (*0, µ_1_*) → (*2, µ_1_*) → (*0, µ_2_*), …, (・, ・). The observer’s states are represented as (sensor state, memory state). The sensor changes from its basal state (*0*) to another state (*1, 2, …*) by measurement. After ICM measurement, the sensor returns to the basal state *0*. Measurements are recorded in the memory, such as *µ_0_, µ_1_, µ_2_.* (**b**) The observer’s state transition is modified to obtain a state transition synchronizing with the system, such as (*0, µ_0_*) → (*0, µ_1_*) → (*0, µ_2_*), by removing intermediate sensor-states that are not in the basal state.

In Figure 6a, the sensor changes state from (*0*, *μ_0_*) to (*1*, *μ_0_*) by cognizing the system in state *s_1_*, which is a discriminative cognition and also functions as an ICM measurement. In this process, the memory now cognizes the sensor in state 1 and changes from *μ_0_* to *μ_1_*. The sensor then returns to the basal state *0*, and the entire state of the observer becomes (0, *μ_1_*). This observer’s cognition is represented by clarifying the intermediate state, (*1*, *μ_0_*) between (*0*, *μ_0_*) to (*0*, *μ_1_*), as shown in Figure 6a. If the intermediate states between (*0*, *μ_i_*) and (*0*, *μ_i+1_*) are concealed, the process is obtained as shown in Figure 6b. Cognition by an EO determines a unit time, and therefore the timescale of the observed system. 

The above example uses the case of an external observer, but the same treatment can be applied for internal observers when interactions occur between a focal observing cognizer and an object system (i.e., the environment of the cognizer). When focusing on living systems with a certain type of internal cognizer (observers) equipped with sensors and memories, the above model may be useful compared to the abstract version of the CS model used in Section 3 and Section 5. The above argument using the inverse-causality model indicates that mathematical formalism of the ICM/W processes and that of the CS model are quite compatible with each other.

## 8. Conclusions

The information concept is often used without explicit definition, usually reified as if it is a material entity, and sometimes confused with the amount of information. In this paper, “information” is defined as the related state-change, which is nothing other than “cognition” in the CS model. The cognition concept unifies the epistemic state-changes for observers and the ontic state-changes for material entities in the framework of the CS model, through which the issue of the E-O duality can be resolved (Section 3 and Section 4).

By using this framework, four types of probability (Section 5) and four types of entropy as a measure of the probability distribution (Section 6) are elucidated. The different interpretations of the same mathematical formulation of entropy and those of the amount of information, due to the differences between the four probability types, cause controversies in physics and biology. Scientific investigations would remain in a conceptual mess if different concepts of entropy and information, as discussed separately in Section 6, were not differentiated clearly under the same mathematical formulae in the literature. Based on the framework presented in this paper, a detailed discussion of specific controversies in various research fields is required in the future.

Lastly, the E-O circularity has been addressed from an internalist model in which only a temporal sequence of data (percepts) are assumed (i.e., the inverse-causality model; Section 7). Information in this internalist model is not identical to “cognition” in the CS model because this model assumes the existence of entities (cognizers) outside a focal cognizer. In the inverse-causality model, the inverse-causality process (i.e., the contrapositive of the statement of the principle of causality) generates symbols referring to external entities, which is the it-from-bit type of information. The inverse causality corresponds to measurement (distinction) of different states of reality in the CS model (an externalist model). It is suggested that the inverse causality process can be incorporated into the CS model (Section 7.3). A certain kind of cognizer can perform this kind of data processing to constitute symbols referring to the external and build an internal model for the external reality. Therefore, the mathematical formalism of the inverse causality processes and the CS model are compatible with each other. 

## Figures and Tables

**Table 1:** Four types of probability depending on the cognizer (observer) and on conditions for determination.

	External Cognizer	Internal Cognizer
Determined under a particular cognition	External P_cog_	Internal P_cog_
Determined under overall cognitions	External P_overall_	Internal P_overall_

## Appendix A. Deterministic Formalization in the Cognizers-System Model

Determinism can be formalized using the cognizers-system model. Deterministic systems obey the principle of causality. The principle postulates that given a state of the world (i.e., the whole cognizers system) at any point in time, the successor state is uniquely determined by both the world state and the world property represented as the motion function (*F*). In other words, given that two states (*u_i_* and *u_j_*) of the world are the same, i.e., *u_i_* = *u_j_*, then their successor states are the same, i.e., *F*(*u_i_*) = *F*(*u_j_*):

If *u_i_* = *u_j_*, then *F*(*u_i_*) = *F*(*u_j_*). (A1)

Consider the two-cognizers system of a focal cognizer, *C*, and its environment, *E*, with motion functions *f_C_* and *f_E_*, respectively. Here, *f_C_*: **U** → **C**, *f_E_*: **U** → **E**, where **C**, **E**, and **U** (= **C** × **E**) are the state spaces of the focal cognizer (*C*), the cognizer’s environment (*E*), and the world (*U*), respectively. *F*(*u_i_*) can then be expressed as (*f_C_* (*u_i_*), *f_E_* (*u_i_*)), where *u_i_* = (*c_i_*, *e_i_*). Designating (*f_C_*(*u_i_*), *f_E_*(*u_i_*)) as (*f_C_*, *f_E_*) (*u_i_*) = *F*(*u_i_*), where (*f_C_*, *f_E_*) () is denoted as *F*(). *f_C_* determines one successor state of *C*, *c_j_* uniquely in terms of a given world state, *u_i_* (= (*c_i_*, *e_i_*)). This transition is expressed as *f_C_*(*c_i_*, *e_i_*) = *c_j_*. Similarly, *f_E_* determines a successor state of *E*, *e_j_*, uniquely in terms of a given world state, which is expressed as *f_E_*(*c_i_*, *e_i_*) = *e_j_* (*c_i_*, *c_j_* ∈ **C**, *e_i_*, *e_j_* ∈ **E**). As such, the causal principle is obtained in terms of cognizers.

If *u_i_* = *u_j_*, then *f_C_*(*u_i_*) = *f_C_*(*u_j_*); if *u_i_* = *u_j_*, then *f_E_*(*u_i_*) = *f_E_*(*u_j_*), (A2)

where *u_i_* = (*c_i_, e_i_*), and *u_j_* = (*c_j_, e_j_*)

Equation (A2) is equivalent to Equation (A1) because Equation (A2) can be expressed as: if *u_i_* = *u_j_*, then (*f_C_*(*u_i_*), *f_E_*(*u_i_*)) = (*f_C_*(*u_j_*), *f_E_*(*u_j_*)), where (*f_C_*(*u_i_*), *f_E_*(*u_i_*)) = *F*(*u_i_*), and (*f_C_*(*u_j_*), *f_E_*(*u_j_*)) = *F*(*u_j_*) by definition.

Taking the contraposition of Equation (A1), the causal principle is obtained in another form:

If *F*(*u_i_*) ≠ *F*(*u_j_*), then *u_i_* ≠ *u_j_*. (A3)

This implies that given two different states occurring at two different points in time, then their preceding states are also different. Note that Equation (A3) does not imply that if *u_i_* ≠ *u_j_,* then *F*(*u_i_*) ≠ *F*(*u_j_*). In other words, the same world state may proceed from different previous states without violating the causal principle.

The above causal principle can again be expressed equivalently in terms of motion functions of the cognizers, as follows. Taking the contraposition of Equation (A2), the followings are obtained:

If *f_C_*(*u_i_*) ≠ *f_C_*(*u_j_*), then *u_i_* ≠ *u_j_*; if *f_E_*(*u_i_*) ≠ *f_E_*(*u_j_*), then *u_i_* ≠ *u_j_*. (A4)

Equation (A4) is equivalent to Equation (A3) because Equation (A4) can be expressed as: If *f_C_*(*u_i_*), *f_E_*(*u_i_*)) ≠ (*f_C_*(*u_j_*), *f_E_*(*u_j_*)), then *u_i_* ≠ *u_j_*. Again note that it is not necessarily true that if *u_i_* ≠ *u_j_*, then *f_C_*(*u_i_*) ≠ *f_C_*(*u_j_*) and that if *u_i_* ≠ *u_j_*, then *f_E_*(*u_i_*) ≠ *f_E_*(*u_j_*). It is possible that *f_C_*(*u_i_*) = *f_C_*(*u_j_*) when *u_i_* ≠ *u_j_*, without violating the causal principle. When both *C* and *E* change respectively to the same state in terms of the different states of *U*, *u_i_*, and *u_j_*, then the world arrives at the same state from different states. At this point in time, two-to-one mapping occurs, which does not violate determinism, that is, the causal principle Equations (A1) or (A3). However, according to this principle, the world will follow the same path after arriving at the same state.

## Appendix B. Pattern as Relation

In the set theory, a particular relation among elements of *k* sets is represented as a subset of the direct product of the *k* sets. A particular pattern (or structure, organization, or order) that is formed by *k* cognizers can be represented in terms of a relation among the states of the cognizers under consideration, in which a *k*-ary relation can be defined by a subset of the direct product of *k* state sets (spaces).

Consider a cognizers-system (CS) with state-space **U**, and this system is composed of cognizers, *C_1_, C_2_, …, C_n_*, with state-spaces **C_i_** (1 ≤ *i* ≤ *n*). **U** can be defined as the direct product of all these components, i.e., **U** = ∏ **Ci**. Direct products of state-spaces of *k* component cognizers, e.g., **C_1_** × **C_2_** × **C_3_**, can also be considered by choosing from the set {**C_1_, C_2_, …, C_n_**}. A subset of such a direct product represents a relation between states of cognizers, e.g., *C_1_*, *C_2_*, and *C_3_*. For example, a particular configuration of atoms or molecules forming a polymer molecule can be represented using a subset that defines their positional states in relation to each other.

Given a CS and a set of the states that occurred in a particular period of time under consideration, we can consider the number of states belonging to a particular subset of the direct product of particular cognizers’ state sets as a pattern formed by the component cognizers. The ratio of this number to the total number of states of the CS dynamics in that period of time is the relative frequency of the pattern occurring. Such pattern formation by cognizers can be attained by particular discriminative and selective actions of cognizers [26,27].

The above description of a pattern, in terms of relation, uses the concept of state from the viewpoint of the meta-observer. However, the same description of patterns is possible for EOs and IOs by considering that states of an observed system, or of its part, are constituted (realized) by cognitions.

## Appendix C. General Representation for the Relationship between Pcog and Poverall

The argument in Figure 3 and Figure 4 uses *c_x_* → *c_y_* → *c_z1_* or *c_z2_* as an example of the cognition sequence of an EO or IO, which is extracted from a continuous state sequence, such as: …*c_x_*, *c_y_*, *c_z1_*, …, *c_x_*, *c_y_*, *c_z2_*, …, *c_n_*. Let us consider a general form of a one-to-many correspondence between a given conditional cognition (*c_x_* → *c_y_*) and *N* kinds of resultant cognitions (*c_y_’* → *c_z1_*, *c_z2_*, …, or *c_zN_*). Note that each kind of cognition can occur two or more times in the sequence.

In this general form, the starting state (*c_y_’*) of the second cognitions (results) is not necessarily the same as the end (*c_y_*) of the first cognition (observation). Let us denote *c_x_* → *c_y_* as observation *B_i_*, which may include two or more kinds of cognition, such as *B_1_* and *B_2_*; and, *c_y_’* → *c_zk_* (1 ≤ *k* ≤ *N*) as a resultant event *A_j_* occurring under *B_i_*.

A given sequence of percepts of an EO or IO can be represented, for example, as

*…, B_1_, A_1_, …, B_1_, A_2_, …, B_2_, A_3_, …, B_1_, A_1_, …, B_2_, A_2_, …, B_1_, A_1_, …,*

where ordered pairs (observation, result) are represented, e.g., *(B_1_*, *A_1_)*. P(*A_j_*|*B_i_*), P(*A_j_*), and P(*B_i_*) can be obtained from the sequence. The summation of P(*A_j_*|*B_i_*)P(*B_i_*) for all *B_i_* equals P(*A_j_*).

## Appendix D. Sense Data and the Reality

In the philosophy of perception, sense data indicate percepts that occur immediately in the self or the mind. According to Russel [13] (Chapter 1), sense data are “the things that are immediately known in sensation: Such things as colors, sounds, smells, hardness, roughness, and so on”. In the standard usage of this term, sense data are the kind of thing we are directly (or immediately) aware of in perception, not something derived from direct awareness. For example, when one sees a table, the percept “this table” is not a sense datum, which is a mental idea derived from such sense data as shapes and colors. The idea of “table” is a percept that is indirectly derived from these sense data, occurring in the mind. However, there is no validation that sense data are caused by something existing independently of the self, which can be understood by considering sense data occur in hallucination and dreams [45,46]. In this paper, immediate percepts like sense data and derived data are not distinguished in the internalist model because they differ only in meaning to the self. Solipsism holds that I (the self) alone exists, and others are representations of the self. This proposition cannot be proved to be false. A possible resolution of the issue of E-O circularity is to find a way to constitute representations for the external reality (i.e., “inverse causality” in this paper), which is consistent with the externalist model (i.e., “measurement” in this paper).

## Appendix E. Inverse Causality and (In)Determinism

Whether our universe is deterministic or indeterministic cannot be empirically determined because both are unfalsifiable hypotheses [27]. However, the device-mediated measurement is undeniably an essential process in science, which is evident in the descriptions for Materials and Methods section in publications. Surprisingly, the realization by device-mediated measurement is possible under the assumption that the world fulfills the unique-successor principle, which is equivalent to the contraposition of determinism. It may be true that the future cannot be predicted using data (percepts) from the past because it is impossible to know the motion function of the world *F* (Section 3.2) from a finite amount of past data [47,48]. In other words, the deterministic state-sequence can be constructed only for past events that have already happened. Here, we can have two different stances about determinism of the world model in Section 3. The first stance is that the world (delineated by the square frame in Figure 1) is open to the future [29], which continues to become enlarged by incorporating new entities thus far unknown. The second is that the world has already included everything, including the “would-be-discovered” part, which is described currently as the unknown environment of us. Both are, again, metaphysical assumptions, but it is important to clarify when we debate the issue of (in)determinism.
